# Three-dimensional ultrastructural analysis of human skin with the arrector pili muscle interacting with the hair follicle epithelium

**DOI:** 10.1038/s41598-025-88615-y

**Published:** 2025-02-04

**Authors:** Tomonobu Ezure, Kyoichi Matsuzaki, Hidetoshi Urakubo, Nobuhiko Ohno

**Affiliations:** 1 MIRAI Technology Institute, Shiseido Co., Ltd, Yokohama, Kanagawa Japan; 2 Department of Plastic and Reconstructive Surgery, School of Medicine, International University of Health and Welfare, Narita, Japan; 3 Section of Electron Microscopy, National Institute for Physiological Sciences, Okazaki, Aichi Japan; 4 Division of Ultrastructural Research, National Institute for Physiological Sciences, Okazaki, Aichi Japan

**Keywords:** Serial block-face scanning electron microscopy, Nerve fibers, Arrector pili muscle, Hair follicle, 3-D reconstruction, Anatomy

## Abstract

This study developed a three-dimensional ultrastructural analysis application using serial block-face scanning electron microscopy (SBF-SEM) to investigate surgically acquired human skin tissues containing the arrector pili muscle. We utilized the en bloc staining, including reduced osmium, thiocarbohydrazide, and lead aspartate, as well as the embedding using a carbon-based conductive resin. Next, we obtained serial images with SBF-SEM. The results revealed dense nerve fiber networks branching from nearby nerve fiber bundles outside the muscle and running among muscle fibers. Additionally, the dense nerve network running through and along arrector pili muscle fibers rarely penetrates the connective tissues between smooth muscle fibers and epithelial cells. Furthermore, in the observation area, no individual smooth muscle fibers formed adhesion structures with the epithelial cells of the hair follicle, ending in the dermal extracellular matrix near the epithelial cells. These results indicate the usefulness of this approach for three-dimensional ultrastructural analyses of human skin tissues comprising follicular units and revealing structural changes in skin tissues, especially the arrector pili muscle and nerve fibers with hair follicular epithelium, in aging and diseased conditions.

**Supplementary Information:**

The online version contains supplementary material available at 10.1038/s41598-025-88615-y.

## Introduction

The skin is the body’s outermost layer, functioning as boundary between the body and the environment. Additionally, it contains several systems that act to maintain homeostasis^1^. For example, sebaceous and sweat glands secrete sebum and sweat, respectively, moisturizing the skin surface, helping regulate the body’s temperature, and contributing to the skin’s barrier function against microorganisms such as bacteria^2^. Furthermore, fine or vellus hairs, produced by hair follicles, cover the body surface and can respond to the external environment^3^.

Hair muscles, also known as arrector pili muscles (APM), are smooth muscles extending from the connective tissue around the hair follicles to the skin surface around the papillary dermis^4^. These muscles contract in response to emotional and cold stimulation, causing the fine hairs to rise and goose bumps to form. In hairy animals, the raised hairs hold air to maintain the body temperature, but the importance of this muscle is poorly understood in humans. Based on its morphology as a pilosebaceous unit (hair follicle, sebaceous gland, and APM), the APM is considered to maintain the hair at a particular position to act as a physical sensor^5^. Further, as the APM runs under the sebaceous gland, the APM may squeeze the sebaceous gland to secrete sebum^6^. Hair follicle stem cells are located in the bulge area adjacent to the APM^7^. However, the APM morphology, especially whether and how it contacts the hair, remains unclear due to the difficulty of observing this complex three-dimensional (3D) organ in situ.

We previously reported that a single APM of facial skin containing vellus hairs had 1.6 branches, was located 943.6 μm from the skin surface at a 28.8-degree angle, and had 1,657.9-µm length and mean volume of approximately 20-million µm^3^ based on X-ray micro-computed tomography analyses^8^. However, the limited resolution (on the micron order) made it difficult to assess the status of individual muscle fibers and their connection to adjacent areas. Furthermore, the innervation system of this muscle, which enables quick response to stimulation, also remains unclear, although androgenic nerve fibers controlled APM^9^.

This study developed an application of the 3D ultrastructural analysis using SBF-SEM to examine surgically acquired human facial skin tissues containing vellus hairs and APM and to address the above questions. The present results indicate that our 3D ultrastructural analysis of human skin tissues can help reveal the unexplored ultrastructural characteristics of the APM, including their interactions with nerve fibers and the follicular epithelium.

## Results

**Fig. 1:**
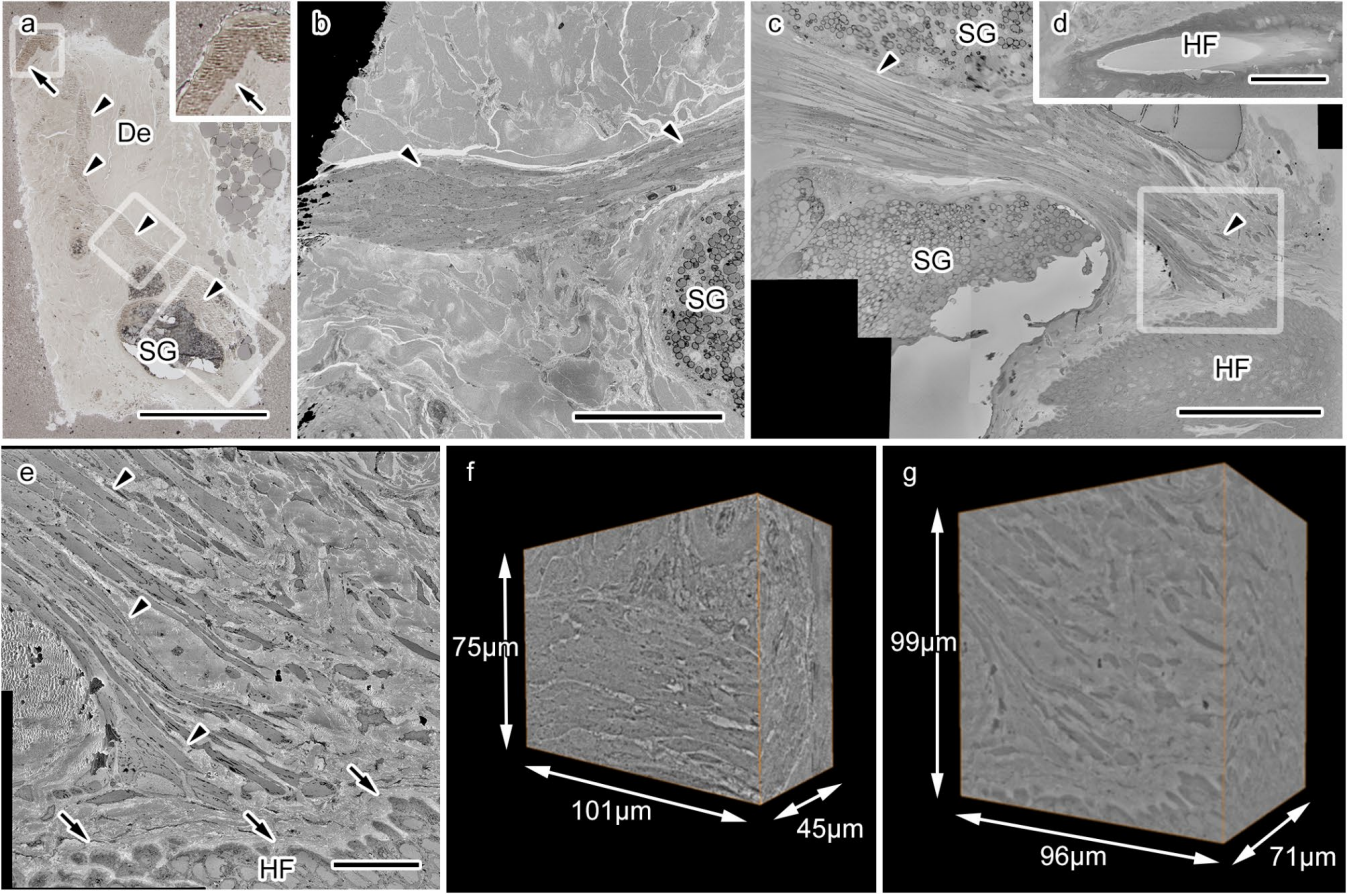
Acquiring serial electron microscopic images from the human arrector pili muscle (APM) with serial block-face scanning electron microscopy. (**a**) A light microscopy image of the dermis (De), including the APM (arrowheads), ending close the an epidermis (arrow). The area labeled with a small rectangle is magnified in the inset, and the areas indicated by medium and large rectangles are magnified in panels (**b**) and (**c**), respectively. SG: sebaceous gland. (**b**) An electron microscopic image showing the middle region of the APM (arrowheads). (**c**) An electron microscopic image showing the area near the end of the APM (arrowheads), approaching the hair follicle (HF) epithelium. The area indicated by a rectangle is magnified in (**e**). (**d**) An image of the HF pore in (**c**), obtained from another slice of the serial images. (**e**) A highly magnified image of the APM tip near the HF demonstrating that muscle fibers (arrowheads) extend close to the HF epithelium. (**f**) A three-dimensional (3D) reconstruction of the dataset of serial images at high resolution acquired from the middle region of the APM, covering about 101 × 75 × 45 μm^3^. (**g**) A 3D reconstruction of the dataset of serial images at high resolution acquired from the end of the APM, covering about 96 × 99 × 71 μm^3^. Scale bars: 500 μm (**a**), 100 μm (**b** and **d**), or 20 μm (**c**,** e**, and** f**).

We first obtained thick (~ 1 μm) sections from multiple blocks of embedded human skin tissues from one patient and found APM which ran in dermal areas and was located near the epidermis and sebaceous glands (Fig. 1a). The APM was divided into three regions of interest (ROI), and each of them was similarly examined by imaging, since the entire APM was too large for SBF-SEM. The analysis excluded the area near the epidermis due to the insufficient volume of the remaining muscle tissue. The location of the middle region of the APM was determined by matching to light micrographs, using the general morphological features of the APM and the adjacent sebaceous gland as landmarks (Fig. 1a-b). The last ROI contained the APM with the surrounding sebaceous gland and the APM tip interacted with epithelial cells (Fig. 1a, c), later identified as a part of the hair follicle in an image acquired at a lower magnification (Fig. 1d). Due to the limited observation volume in our dataset, we could not identify the phase of the follicle cycle. However, the close apposition of the APM tip and epithelial cells was clearer and the distance between individual APM fibers and the hair follicle epithelium could be measured in high-resolution images (Fig. 1e). We acquired a dataset of serial images at high resolution covering about 101 × 75 × 45 μm^3^ from the middle part of the APM (Fig. 1f) and about 96 × 99 × 71 μm^3^ from the tip part of the APM (Fig. 1g). Both datasets included many APM fibers with their surrounding nerve fibers; thus, subsequent analyses utilized these two sets of serial images.

**Fig. 2:**
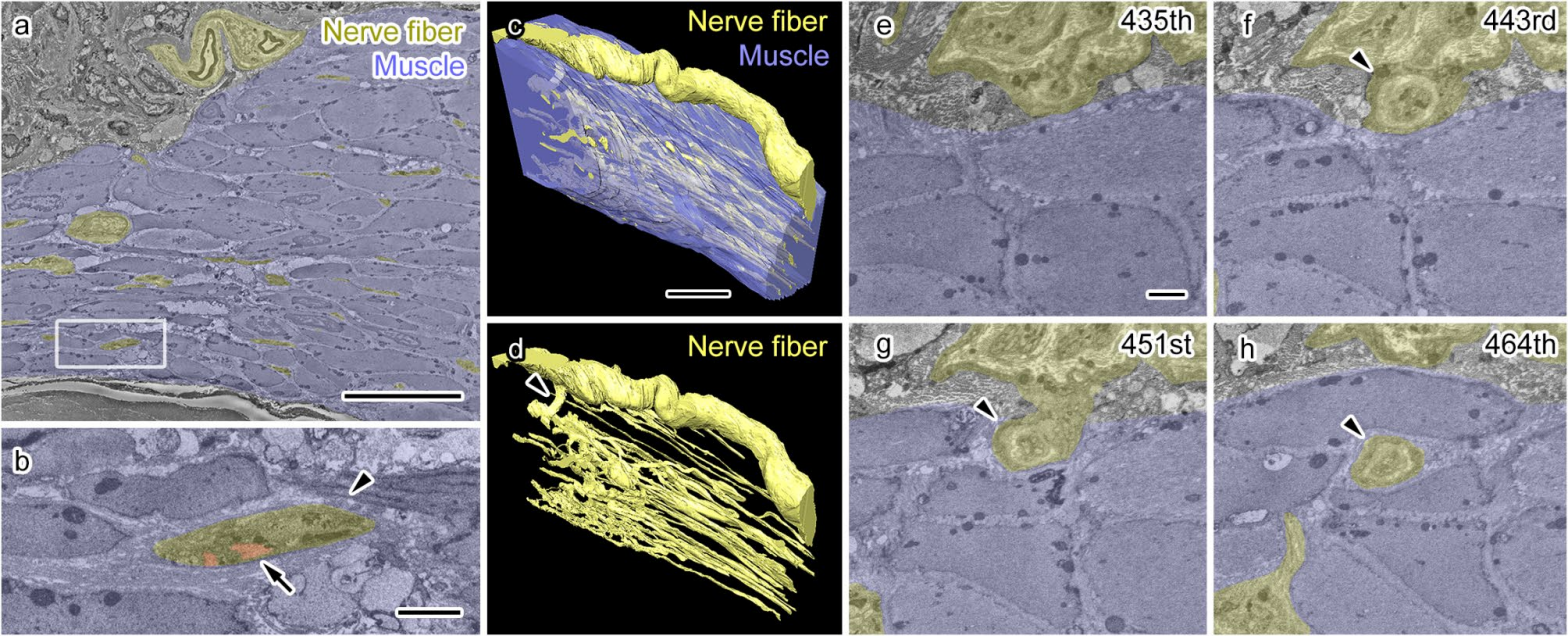
Dense nerve fiber network in the middle of the arrector pili muscle (APM). (**a**,** b**) Representative electron micrographs with segmentation depicting an APM bundle (blue) and nerve fibers (yellow). The area indicated by a rectangle (**a**) is magnified in (**b**). A nerve fiber (b, arrowhead) containing axonal profiles (**b**, red, arrow). (**c**,** d**) Three-dimensional (3D) reconstruction showing the APM fiber bundle (**c**, blue) and nerve fibers (**c**,** d**, yellow) demonstrate a dense nerve fiber network running throughout the muscle fiber bundle and connected to an adjacent thick nerve fiber (d, arrowhead). (**e**–**h**) Serial electron microscopic images of the nerve fiber connecting the extrinsic nerve fiber and the nerve network inside the APM (**f**–**h**, arrowheads). The number of each image in the serial image dataset is shown in the upper right corner. Scale bars: 20 μm (**a**,** c**), 2 μm (**b**,** e**).

We first analyzed the middle region. Further, the APM as a whole and the nerve fibers in serial EM images were manually segmented and 3D reconstructed (Fig. 2). In all slices, nerve fibers were found mostly in the APM fiber bundle (Fig. 2a, Supplementary Fig. 1a) with axons inside the nerve fibers ensheathed by Schwann cells (Fig. 2b, Supplementary Fig. 1b). Axonal profiles changed their diameters during their trajectory and following individual axons throughout the dataset was impossible; thus, we segmented and 3D reconstructed nerve fibers containing both axons and ensheathing Schwann cells (Fig. 2a-d, Supplementary Fig. 1c, d). Interestingly, nerve fibers were abundant inside the area where APM muscle fibers were forming thick bundles, with a very small number found outside the APM (Fig. 2c, d, Supplementary Fig. 1c, d). Further, we observed connections between the nerve network in the APM and an adjacent thick nerve fiber bundle, which included a myelinated axon surrounded by a thick electron-dense compact myelin and ran through the dermis, when these fibers were followed throughout the dataset (Fig. 2c-h). This connection comprised axons and Schwann cells and penetrated deep into the APM to connect to the nerve fiber networks in the APM (Fig. 2e-h). These results indicate that the nerve network composed of unmyelinated nerve fibers is derived from adjacent dermal nerve fibers and forms a dense network inside but not outside or surface of the middle part of APM.

**Fig. 3:**
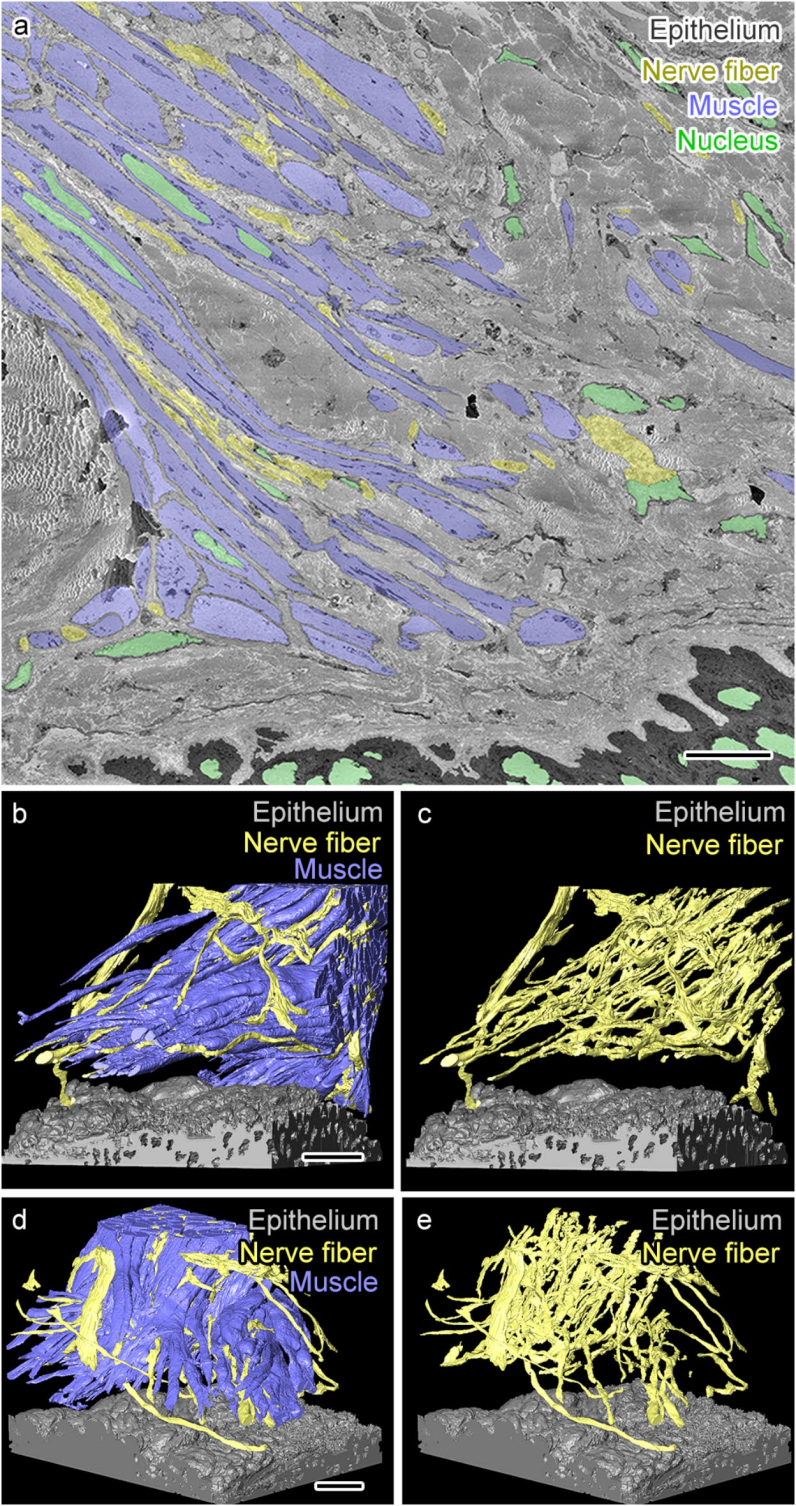
A dense nerve fiber network penetrates and runs along arrector pili muscle (APM) fibers but rarely contacts the hair follicle epithelium. (**a**) A representative electron micrograph with segmentation showing APM (blue), nerve fibers (yellow), hair follicle epithelium (black), and cellular nuclei (green). (**b**–**e**) Three-dimensional (3D) reconstructions of muscle, nerve fibers, and the epithelium from two different angles showing few contacts between nerve fibers and the hair follicule epithelium (**b**,** c**). In contrast, the nerve fiber network runs along the APM (**d**,** e**). Scale bars: 10 μm.

Next, we investigated the serial EM image dataset including close apposition between the APM and the hair follicle (Figs. 1e and g and 3a). Through efficient segmentation with UNI-EM, we performed segmentation of the individual APM fibers among which nerve fibers were running (Fig. 3a), but not segmentation of the APM as a whole, as shown in Fig. 2. We then performed a 3D reconstruction to carefully examine the distribution of nerve fibers. This improvement enabled visualization of the more detailed trajectory of individual APM fibers and nerve fibers (Fig. 3b-e). This analysis revealed that nerve fibers form a network that follows the trajectory of muscle fibers and divides alongside the bifurcating muscle fiber bundles (Fig. 3b-e). The nerve fiber network did not prominently extend outside the APM, as observed in the middle part of APM (Fig. 2), with very few nerve fibers found outside the APM. Another interesting characteristic was the end of the APM, which showed no direct contact with the hair follicle, and few direct contacts between nerve fibers and the hair follicle epithelium (Fig. 3b-e). These results indicate a consistent nerve network inside the middle and deep parts of the APM, and the area of the APM interacting with the hair follicle has some gaps where direct interactions between the APM nerve fibers and the hair follicle epithelium are not clearly observed.

**Fig. 4:**
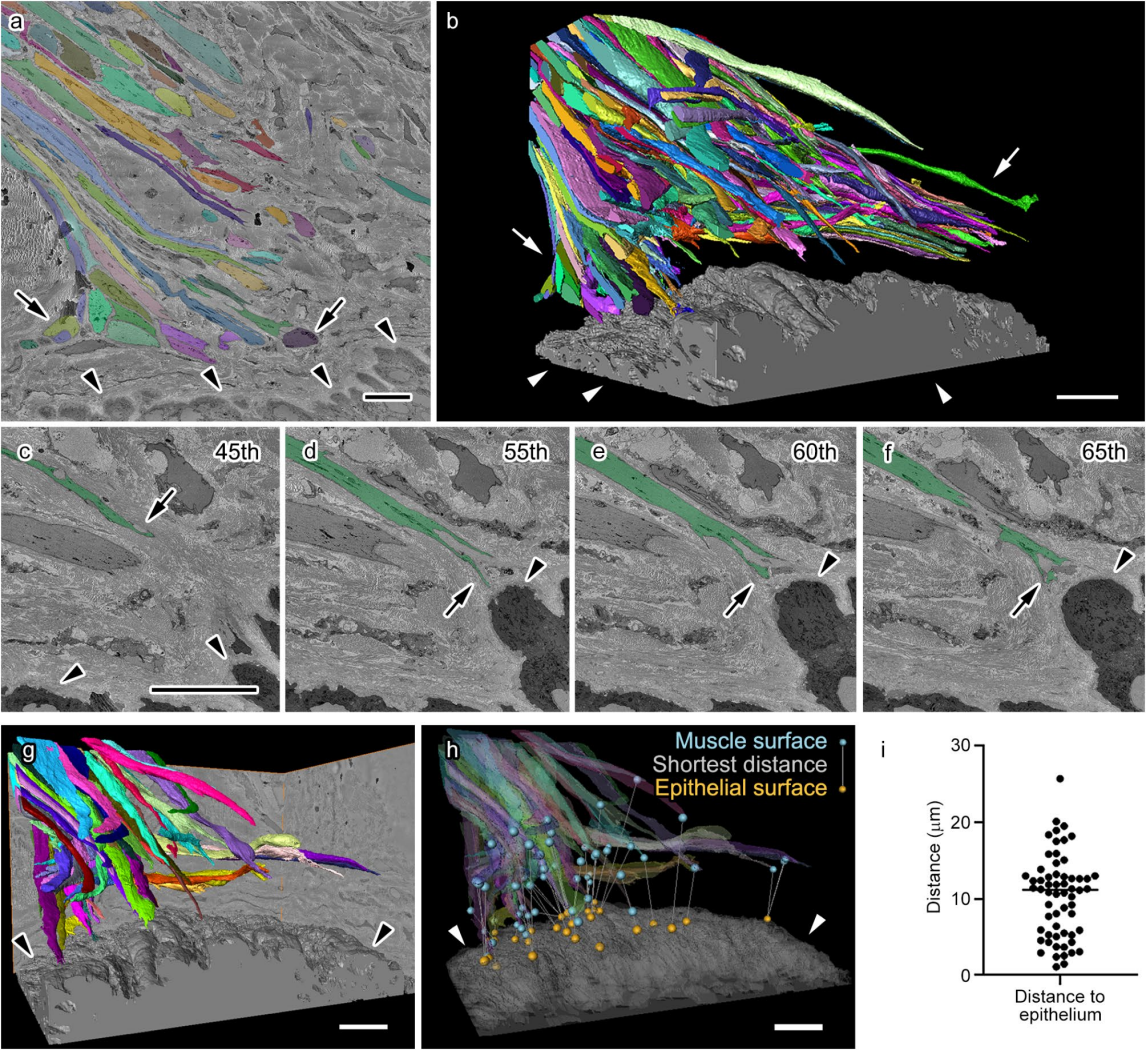
Interaction between the arrector pili muscle (APM) and connective tissues surrounding the hair follicle epithelium. (**a**) A representative image of segmentation showing all individual APM fibers (arrows, various colors) and the hair follicle epithelium (arrowheads, gray). (**b**) Three-dimensional (3D) reconstruction of individual APM fibers (arrows, various colors) and the hair follicle epithelium (arrowheads, gray). Note that each fiber appears to have a variable but non-negligible distance from the hair follicle epithelium. (**c**–**f**) Serial images showing an individual muscle fiber (arrows, green) and the epithelium (arrowheads, black). The number of each image in the serial image dataset is shown in the upper right corner. (**g**,** h**) 3D reconstruction showing only muscle fibers whose ends are included in the observed volume (various colors) and being close to the epithelium (arrowheads, gray) is shown together with the closest distance between each fiber and the epithelium (**h**, lines with blue spheres on the muscle side and orange spheres on the epithelium side). The muscle surface closest to the epithelial surface is not always at the end of the muscle. (**i**) Graph illustrating the shortest distance between each muscle fiber and the epithelium. Each dot represents single APM fiber. Scale bars: 10 μm.

The interactions between APM and the hair follicle epithelium play important roles in the function of hair follicular stem cells^10^; thus, we carefully investigated the connection between APM and the hair follicle epithelium (Fig. 4). APM segmentation data was further processed to allow individual APM fiber isolation (Fig. 4a, b) and subsequent measurement of its morphological parameters. This analysis revealed relatively large gaps between the APM and the hair follicle epithelium (Fig. 4c-f). Excluding APM fibers truncated at the edge of the dataset (Fig. 4g, h) confirmed the lack of contact between the end of APM fibers and the hair follicle epithelium; rather, they contacted the dense extracellular matrix in dermal areas (Fig. 4c-f), which was partially adjacent to the hair follicle epithelium (Fig. 4c-f). In addition, this data showed that the surface of muscle fibers closest to the epithelial surface is not always at the tip of muscle fibers (Fig. 4g, h). There was a median distance of approximately 10 μm between the surface of APM fibers and the epithelium (Fig. 4i). These results indicate that APM forms a distant interaction with hair follicles and the interaction could be mediated by the dense collagen network located between the APM and hair follicles.

## Discussion

This study applied 3D ultrastructural analysis using SBF-SEM to investigate the human skin tissue and the spatial distribution and interactions of APM, nerve fibers, and follicular epithelium at the ultrastructural level. Our findings revealed that nerve fibers formed dense networks branching from nearby dermal nerve bundles and running among APM fibers, with rare connective tissue penetration surrounding them. APM fibers did not form adhesion structures with hair follicle epithelial cells but ended in the dermal extracellular matrix. These results indicate the usefulness of this approach for the 3D ultrastructural analysis of human skin tissues and their follicular units and the potential for revealing changes in APM and nerve fibers associated with hair follicles in aging and diseases.

To establish Digital-3D skin technology, we previously observed APMs with microCT and quantitatively evaluated the structure of vellus hair muscles^8^. However, such observation approaches and common optical microscopy are frequently used to identify the proximity of APM and related structures, but the spatial associations of these structures at the ultrastructural level are difficult to confirm with approaches other than electron microscopy. In addition, conventional 2D observation hinders precise distance and interaction measurements among these structures. A previous study showing the 3D morphology and topology of keratinocytes and melanocytes in senile lentigo using 3D electron microscopy^11^ allowed to visualize all membrane connections and corresponding distances as well as insights into the regulation of physical interactions among these structures under physiological and pathological conditions. This study paved the way for future 3D ultrastructural analysis, providing unique information into the structural regulation of APM along with associated nerve fibers and follicular epithelium in human skin, despite the limitations of electron microscopy, such as the limited sizes of the observable areas and fewer options for molecular imaging^12^.

This study revealed that the nerve branches of the dermal nerve fiber bundle formed dense networks in APM but hardly contacted the epithelial cells of hair follicles. Skin innervation, including sensory and autonomic nerve fibers, is critical for skin functions such as sensory perception^13^. Particularly, sympathetic innervation in the APM plays a pivotal role in hair functions, including hair growth and deficit^14^. Our ultrastructural analyses using serial EM images revealed that such innervations primarily composed of unmyelinated nerve fibers are supported by nearby dermal nerve bundles, including thinly myelinated A-delta fibers and unmyelinated C-fibers of sensory and postganglionic autonomic nerves^15^. The nerve network and its role have been extensively investigated in the area of close APM and hair follicle interaction, known as bulge^10^. While these nerve fibers control APM function, they also play another important role. Previous research in mice provided evidence supporting the concept that the adrenergic nerves and stem cells in hair follicles are physically connected and the stem cell activity is regulated through norepinephrine secretion from the sympathetic nerves^16^. This study has limitations, such as its observational nature and the unknown follicle cycle stage; thus, this suggests a dynamic regulatory system in humans where physical connections, similar to those in mice, may be formed during specific phases of the hair follicle cycle. Additionally, there may be differences in physical connections among species or body areas as the current study focused on the vellus hairs in human facial skin. Future studies are warranted to address these possibilities and understand the interactions between nerve branches of the APM and the hair follicles.

This study revealed the connections of all individual APM fibers to the dermal extracellular matrix near the follicular epithelial cells and observed no adhesion structures between the epithelial cells of the hair follicle and APM. Previous studies indicated physical connections between APM and the bulge of hair follicles via basement membranes, which may be crucial for stem cell function^10^. Previous research in fetal skin indicated hemidesmosome-like interaction between APM and the basement membrane^17^. A direct interaction may be more evident at early developmental stages than in older humans, being also modulated by the hair cycle. Understanding variations and/or changes in the interactions between APM and hair follicles may help understand hair pathology and develop effective solutions to hair diseases.

## Materials and methods

### Sample collection and fixation

The ethics committees of the Shiseido Research Center, the International University of Health and Welfare, and the National Institute for Physiological Sciences approved this study (C02208, 23-Nr-051, EC01-060), which was conducted in compliance with the ethical guidelines for life science and medical research involving human subjects. After obtaining informed consent, a human skin sample, facial skin containing vellus hairs, was prepared from the surplus forehead skin excised during plastic surgery on a 20-year-old woman. The sample was fixed by immersion in 2.5% glutaraldehyde and 2% paraformaldehyde in 0.1 M phosphate buffer over a few nights. The sample was immersed in the fixative as soon after excision as possible, and the time between excision and immersion was about 10 min. The sample size was approximately 2–3 mm.

## En bloc staining, embedding and imaging

The sample was stained and embedded in the resin as previously described with some modifications^18^. Briefly, the tissues were washed four times with PBS for 3 min after cutting the sample into small pieces, and sequentially incubated with 2% OsO4 in 1.5% potassium ferrocyanide for 60 min at 4℃, filtered 1% thiocarbohydrazide solution for 20 min at room temperature (RT), 2% OsO4 for 30 min at RT, 2% uranyl acetate overnight at 4℃, and a 0.66% lead aspartate solution at 50 °C for 2 h. Samples were washed with double distilled water after each incubation. Then, the tissues were dehydrated in a graded ethanol series (60%, 80%, 90%, and 95% for 5 min each), infiltrated with acetone dehydrated with a molecular sieve, a 1:1 mixture of resin (Durcupan) and acetone, and 100% resin. The resin was prepared as per manufacturer’s instructions. Upon embedding, 5% Ketjen black was added to increase the resin conductivity^19^. After curing, the resin blocks were transferred onto rivets for imaging, and ~ 1 μm thick sections from each block were transferred to glass slides for light microscopic observation with an optical microscope (BX-63; Olympus Japan Co.). The block containing the identified APM was trimmed, and its surface was treated with gold sputtering to increase block conductivity. The block was then imaged with a field-emission scanning electron microscope (Merlin, Carl Zeiss, with Gemini II column and field-emission electron gun) equipped with 3View, Digital Micrograph (version 3.21.1374.0), and OnPoint detector (Gatan, Inc.) but not with focal charge compensation. The acceleration voltage was 1.2 kV, and the dwelling time was 0.8 µs/pixel. We acquired 561 serial EM images at a 6.5 nm/pixel resolution and 80 nm/slice thickness from the middle region of the APM, and 1,014 serial EM images at a 7 nm/pixel resolution and 70 nm/slice thickness from the APM end interacting with the hair follicle.

## Image analyses

The obtained serial images were analyzed using ImageJ with Fiji plugins (http://fiji.sc/). Contrast adjustment, stitching and alignment were performed with TrakEM2^20^. Manual segmentation and image analyses were conducted using the Microscopy Image Browser (version 2.70, http://mib.helsinki.fi.)^21^, VAST Lite software (version 1.4.0)^22^ and Amira (version 2020.1, FEI Visualization Science Group). UNI-EM (version 0.92), a unified environment for CNN-based automated segmentation of EM images^23^, was used for automatic segmentation of the APM and nerve fibers, followed by manual proofreading. Myelination was determined by identification of electron-dense compact myelin surrounding axons^24^. The linear distances between each APM fiber and epithelial cells were measured using previously described software^24^, which measures the minimal distance between the surfaces of object pairs.

## Electronic supplementary material

Below is the link to the electronic supplementary material.

Supplementary Material 1

## Data Availability

All study data are included in the article.
