# Supplementary material for: Three-dimensional ultrastructural analysis of human skin with the arrector pili muscle interacting with the hair follicle epithelium
Source: Sci Rep. 2025 Feb 4;15:4195. doi: 10.1038/s41598-025-88615-y (PMC11794863; doi:10.1038/s41598-025-88615-y)
Supplement: Supplementary file 1 — Supplementary Material 1 [file 41598_2025_88615_MOESM1_ESM.pdf]

Supplementary Materials

Title: Three-dimensional ultrastructural analyses of human skin with arrector pili muscle interacting with hair follicle epithelium

Authors: Tomonobu Ezure, Kyoichi Matsuzaki, Urakubo Hidetoshi, Nobuhiko Ohno

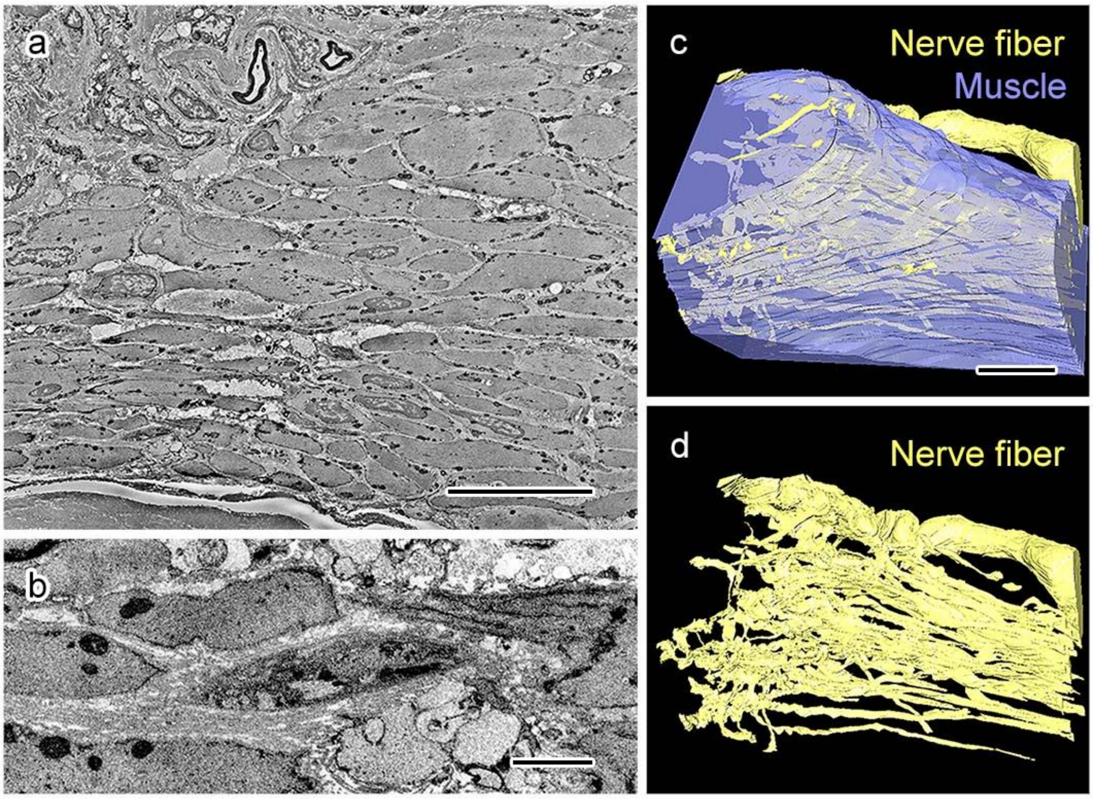

Supplementary Figure 1.

Unsegmented electron microscopic images and additional 3D reconstruction images of the middle region of the arrector pili muscle shown in Figure 2. (a, b) Electron microscopic images at low (a) and high (b) magnifications without coloring of segmentation shown in Figure 2 (Fig. 2a, b). (c, d) Three-dimensional reconstruction of the arrector pili muscle shown in Figure 2 (Fig. 2c, d), which is viewed from another angle and depicting an APM bundle (blue) and nerve fibers (yellow). Scale bars: 20 μm (a, c), 2 μm (b).
